# Multi-Pattern Visual Statistical Learning in Monolinguals and Bilinguals

**DOI:** 10.3389/fpsyg.2019.00204

**Published:** 2019-02-07

**Authors:** Federica Bulgarelli, Laura Bosch, Daniel J. Weiss

**Affiliations:** ^1^Department of Psychology and Program in Linguistics, Pennsylvania State University, University Park, PA, United States; ^2^Department of Psychology and Neuroscience, Duke University, Durham, NC, United States; ^3^Department of Cognition, Development and Educational Psychology, Institut de Neurociències, Universitat de Barcelona, Barcelona, Spain

**Keywords:** statistical learning, visual statistical learning, bilingualism, multi-level statistical learning, language experience

## Abstract

To date, the impact of bilingualism on statistical learning remains unclear. Here we test a novel visual statistical learning task that affords simultaneous learning of two types of regularities: co-occurrence regularities between pairs of elements and the co-occurrence of visual features that could define categories. We compared performance by English monolinguals, Spanish-Catalan bilinguals and Spanish-English bilinguals, as previous studies have suggested that bilinguals might be more open than monolinguals to the presence of multiple regularities, though no previous studies have tested the learning of multiple patterns within a single task. We demonstrated that both monolingual and bilingual participants could learn the co-occurrence probabilities and the features that define categories. To the best of our knowledge, this study is the first to demonstrate that learners can extract co-occurrence regularities along two dimensions in the visual modality. However, we did not detect significant differences in performance across groups. We close by discussing the implications for the growing literature on bilingualism and statistical learning.

## Introduction

The benefits of bilingualism are thought to extend beyond linguistic knowledge, impacting a wide variety of cognitive and social abilities across the lifespan (e.g., Bialystok, 1999; Goetz, 2003; Greenberg et al., 2013; though see Paap and Greenberg, 2013. for a dissenting view). As differences in cognitive tasks between monolinguals and bilinguals are thought to arise early in development (e.g., Kovács and Mehler, 2009a,b; Sebastián-Gallés et al., 2012) it is natural to wonder whether bilingual experience impacts statistical learning, the ability to track distributional regularities from sensory input.

## Statistical Learning

Statistical learning is thought to support many aspects of early language learning (e.g., Saffran et al., 1996a; Gomez and Gerken, 1999; Maye et al., 2002; Yu and Smith, 2007; Reeder et al., 2013). While studies of statistical learning have a rather long history (e.g., Reber, 1967) this subfield of research took on greater prominence in the mid-1990’s when it was applied toward solving the challenges of speech segmentation by having learners track transitional probabilities between elements in an artificial speech stream (Saffran et al., 1996a,b). Relevant to our study, subsequent research demonstrated that these statistical learning effects extend to the visual domain, as learners are able to track both temporal probabilities and spatial relationships (e.g., Fiser and Aslin, 2001, 2005). Subsequent research in statistical learning has also tested for relationships that require accumulating associative information across time, such as in cross-situational statistical learning (Yu and Smith, 2007). This task explored the possibility that tracking statistical associations over time could provide leverage in solving referential uncertainty during word learning. The current study explored whether bilingual experience impacts statistical learning when two types of regularities are simultaneously available in the input, including spatial relationships between objects and visual feature co-occurrences within objects.

## Bilingualism and Statistical Learning

To date, statistical learning studies comparing monolinguals and bilinguals have adopted several approaches. Arguably the most straightforward method has been exploring whether bilinguals differ from monolinguals in statistical learning involving a single set of regularities, usually from segmentation, cross-situational word learning, or rule learning tasks. Yim and Rudoy (2013) compared monolingual and Spanish-English bilingual children ranging from 5 to 13 years of age on both a visual and an auditory statistical learning segmentation task. The authors found no differences in performance across groups on either task. By contrast, studies using a cross-situational word learning paradigm have yielded mixed results. In these studies, learners hear words presented in random order while seeing objects appear on a screen and must infer which word maps to which object by tracking co-occurrence probabilities over time (see Yu and Smith, 2007). Escudero et al. (2016) found that simultaneous English-Mandarin bilinguals exhibited overall better word learning abilities relative to English monolinguals. However, using a similar paradigm, Poepsel and Weiss (2016) reported that English monolinguals and late English-Spanish and Mandarin-English bilinguals did not differ in their ability to learn one-to-one mappings between labels and their referents (Poepsel and Weiss, 2016). This discrepancy may be due to the proficiency or age of acquisition of the bilinguals tested in each study, or perhaps subtle differences in methodology, or some combination thereof.

A second approach to determining whether bilinguals differ in their statistical learning abilities involves providing multiple cues to segmentation in the input. For example, Wang and Saffran (2014) tested adult monolingual and bilingual learners using a speech segmentation task on an artificial tonal language that contained transitional probability cues that overlapped with suprasegmental tone cues, jointly indicating the location of word boundaries. The authors tested monolingual English speakers, monolingual Chinese speakers (who have experience using tone contrastively in their native language), Chinese-English bilinguals, and an additional group of bilinguals who did not speak a tonal language. Only the bilingual groups succeeded at segmenting the speech stream, suggesting that experience with a tonal language alone (as experienced by the monolingual Mandarin group) was insufficient for learning. Rather, the authors proposed that the overlapping cues may have distracted monolingual learners, and thus enhanced inhibitory control abilities in bilinguals may have allowed them to ignore the tone cue in order to focus on tracking transitional probabilities. Similarly, Bartolotti et al. (2011) presented participants with two artificial languages in Morse Code, manipulating whether the conditions of learning provided low interference (an additional cue reinforced the statistics) or high interference (an additional cue conflicted with the statistics). While in the high interference condition both groups performed equivalently, in the low interference condition bilinguals demonstrated the ability to integrate across cues or ignore one of the congruent cues to segmentation. In sum, bilinguals may be more successful in statistical learning tasks that involve an inhibitory component.

Given that bilinguals contend with multiple languages, it is possible that they may be advantaged when there is more than a single set of statistics in the input. This advantage seems to be present from an early age, as suggested by work on learning multiple rules by bilingually raised infants (Kovács and Mehler, 2009a,b). This hypothesis has resulted in several studies testing learners’ segmentation ability when presented with two artificial languages (e.g., Weiss et al., 2009). Antovich and Graf Estes (2017) presented 14-month-old infants with two alternating, congruent speech streams. The monolingual infants did not learn either language. Bilingual infants, however, successfully segmented both streams (Antovich and Graf Estes, 2017). Given that the languages were statistically congruent (i.e., the statistics of one language did not interfere with the other), the authors concede that the infants could learn both languages as a single larger language.

In contrast to the infant study by Antovich and Graf Estes (2017), experiments with adults in which two statistically incongruent languages were presented with only a single switch midstream find that both monolinguals and late bilinguals exhibit a primacy effect, learning the first language but not the second (Gebhart et al., 2009; Bogulski, 2013; Bulgarelli and Weiss, 2016). These differences may be due to the inventory of the languages (congruent versus incongruent), the number of switches between the languages, general task differences in measuring performance between infants and adults or the age of acquisition of each language for the bilinguals (see Bulgarelli et al., 2018. for further discussion).

While the results from previous studies suggest that the interplay between bilingualism and statistical learning is complex, several trends can be identified (Weiss et al., 2015; Poepsel and Weiss, 2016; Bulgarelli et al., 2018). One recurring theme is that the core statistical learning abilities appear to be unaffected by experience with more than a single language (see also Park et al., 2017). That is to say, most studies that have investigated statistical learning using artificial speech segmentation, cross-situational statistical learning, and rule learning find that participants, regardless of language background, can track statistics emanating from a single underlying model with equivalent proficiency (e.g., Yim and Rudoy, 2013; Poepsel and Weiss, 2016; though see Escudero et al., 2016. for a possible exception). The conditions that seem to more reliably elicit differences between groups often involve multiple structures (e.g., Poepsel and Weiss, 2016; Antovich and Graf Estes, 2017) or multiple competing cues (e.g., Bartolotti et al., 2011; Wang and Saffran, 2014). Further, earlier exposure to a second language (as in the case of simultaneous or early sequential bilinguals) may facilitate differences in learning (Escudero et al., 2016; Antovich and Graf Estes, 2017).

With these ideas in mind, the present study sought to extend the research comparing monolingual and bilingual learners on learning multiple structures by presenting a task that affords tracking of two regularities simultaneously. To date, the approach for presenting bilingual learners with multiple structures has been sequential, with either one artificial language followed by another (e.g., Weiss et al., 2009; Antovich and Graf Estes, 2017) or successively presented mappings of words to objects (e.g., Poepsel and Weiss, 2016). Given that bilinguals are thought to be advantaged in multitasking (Bialystok et al., 2006; Bialystok, 2012; see Moradzadeh et al., 2015. for a dissenting view), as well as in tasks involving non-selective executive control and divided attention (Festman et al., 2010; Poarch and van Hell, 2012), it is possible that this may translate to better performance on a task involving simultaneous tracking of multiple regularities.

We presented learners with a novel visual task that permitted the simultaneous tracking of different types of co-occurrence statistics, one involving the spatial positioning of adjacent characters, and the other involving features comprising categories of characters. We tested two groups of highly proficient bilinguals whose languages differed in the level of proximity of their phonological, lexical, and morpho-syntactic properties. We compared performance across Spanish-Catalan (closer, more similar languages) and Spanish-English (more distant, less similar languages) bilinguals along with a group of English monolingual participants. By including two bilingual groups with different language profiles, we hoped to minimize the chances that an idiosyncratic feature of a particular bilingual population would influence our conclusions. The use of visual stimuli, rather than linguistic input, ensured that there would be no advantage for any group on the basis of familiar phonological or phonotactic cues. Our participants also completed a working memory task (operational span), which allowed us to gain some insight as to how working memory differences might relate to performance on the experimental task.

## Participants

Twenty-four functionally monolingual English speakers (7 males) whose average age was 19.3 years (*SD* = 1.48) participated in this experiment, along with 23 Spanish-Catalan bilinguals (6 males; mean age 20.22, *SD* = 1.98), and 24 Spanish-English bilinguals (11 males, mean age 25.67, *SD* = 4.84). Monolingual participants were recruited from the Psychology Subject Pool at Pennsylvania State University and received course credit for their participation. Using the Language History Questionnaire, monolinguals rated their English proficiency as a 10 (on a 10-point scale). All but one participant reported exposure to a second language, due to a foreign language requirement at Pennsylvania State University. However, they self-rated their proficiency in their second language at an average of 3.29 (*SD* = 1.33) on the 10-point scale, with no rating above 5. An additional 3 participants were recruited at Pennsylvania State University, but excluded from analysis as they rated their L2 proficiency as above a 6, which was above our conservative cutoff for functional monolingualism (see Poepsel and Weiss, 2016). Spanish-Catalan bilinguals were recruited from the Psychology Subject Pool at the University of Barcelona, in Spain, and received course credit for their participation. They rated their Spanish proficiency as a 9.52 (*SD* = 0.71) and their Catalan proficiency as a 9.78 (*SD* = 0.41). They also reported using both languages daily. Due to foreign language requirements, all Spanish-Catalan bilingual also reported some knowledge of English acquired from formal instruction in school settings, and rated themselves as rather proficient in this non-native language (mean = 6.7, *SD* = 1.33). One additional Spanish-Catalan bilingual was recruited, but excluded from the final sample due to low self-rated proficiency in Catalan. Spanish-English bilinguals were recruited from the Pennsylvania State University and received monetary compensation for their participation. They rated their Spanish proficiency at a 9.79 (*SD* = 0.64) and their English proficiency at a 9.17 (*SD* = 0.75), and also reported using both languages daily. The Spanish-English bilinguals did not consistently report exposure to a third language, although seven of them received formal instruction to French, Portuguese, or Italian, and also rated themselves as rather proficient (mean = 5.83, *SD* = 2.31). An additional Spanish-English participant was recruited but excluded from the analyses for not meeting the language requirements. Our sample sizes are consistent with other recent studies comparing groups of bilingual participants on statistical learning tasks (Wang and Saffran, 2014; Poepsel and Weiss, 2016). All experimental protocols, including procedures for obtaining informed consent, were approved by the Pennsylvania State University and Universitat de Barcelona IRBs.

## Stimuli

**FIGURE 1:**
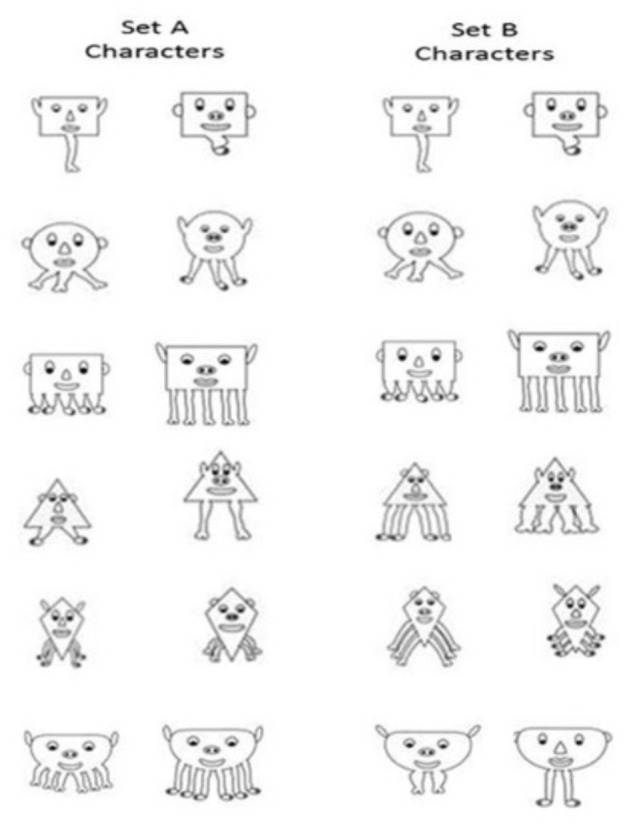
Characters that comprised each Set. The correlated feature (shape and number of legs) varied across Set, and all other features could freely vary.

Using Anime Studio©, we created six classes of characters based on different shapes (triangle, circle, rectangle etc.). Each character class had six features that could vary in addition to the body shape: the shape of the eyes, ears, nose, lips, and feet, as well as the length of the legs. From these features, we created two sets of 12 characters. For Set A, each character shape was paired with a specific number of legs (see Figure 1), such that those two features were always correlated, while the others could freely vary. For example, the circle character was paired with 3 legs and the triangle character with 2 legs. Each set contained two instances of each shape-based character. Every freely varying feature (shape of the eyes, ears, nose, lips and feet, and length of legs) occurred at least once with every other feature (e.g., circle eyes occurred with both types of ears, noses, lips, feet and length of legs) across the entire set of characters (see Figure 1). The second set of characters, Set B, had partial overlap with Set A in that they shared six characters (and thus had the same correlated features of character shape and number of legs). The circle character with 3 legs, and the two rectangles^1^ with 1 leg and 5 legs occurred in both sets. The remainder of the shapes had a different set of correlated features relative to Set A. Similar to Set A, every freely varying feature occurred at least once with every other feature.

**FIGURE 2:**
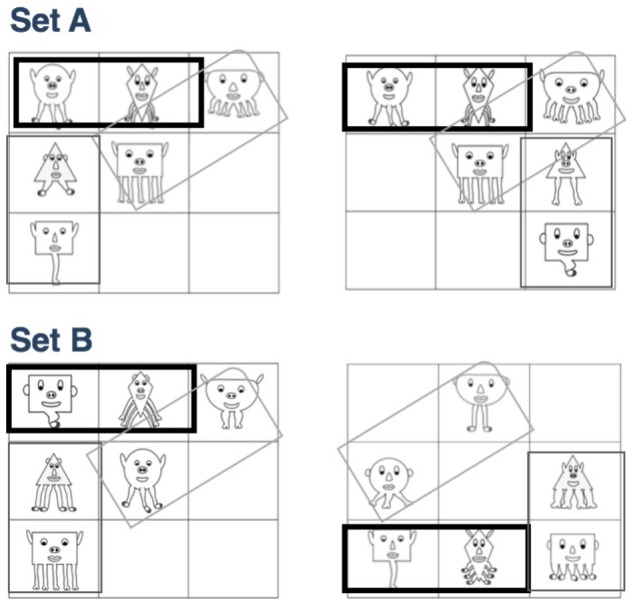
Examples of familiarization scenes for each Set. Horizontal, vertical, and diagonal rectangles demonstrate the statistically congruent pairs.

For the familiarization phase, the characters were spatially organized into visual bigrams on a 3 by 3 grid (see Figure 2 for an example). One bigram always appeared arranged on the horizontal axis (i.e., the circle character next to the diamond character for Set A and the small rectangle character next to the diamond character for Set B; see Figure 2), one bigram always appeared on the vertical axis, and one bigram always appeared on a diagonal. Each type of bigram orientation (horizontal, vertical, or diagonal) could occur in one of two locations (see Figure 2 for the possible options). Each character appeared consistently with one other character. For example, for Set A, one of the circle characters always occurred with the same diamond character. During familiarization, three bigrams were always presented simultaneously (i.e., a total of six characters in every display) with one bigram in each orientation (see Figure 2 for an example of a familiarization scene). Familiarization scenes were created by exhaustively pairing each bigram with the bigrams of different orientations (i.e., the diagonal bigram would be paired with each of the horizontal and vertical bigrams) at each location. Following these criteria, a total of 32 possible scenes were created, which were concatenated and repeated 4 times each during familiarization. The familiarization stream was created by presenting each scene for 3.5 s. The scenes were presented in random order (with the caveat that no scene could repeat itself), with a 500 ms interstimulus interval during which time the screen was black. A separate familiarization stream was created for each set of characters.

**FIGURE 3:**
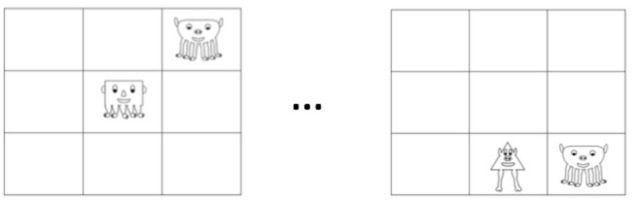
Example of a Segmentation test trial. Each trial consisted of two scenes (presented in succession), with either a pair of statistically congruent shapes, or statistically incongruent shapes. Only one scene was displayed at a time, with order counterbalanced across test trials. After viewing both scenes, participants chose which could have been part of the familiarization video.

Two types of test items were created: one that tested segmentation of the bigrams within the visual scenes (hereafter Segmentation trials, as it required extracting subunits of visual regularities from the larger grid), and one that tested knowledge of the correlated features defining the categories of characters (hereafter Categorization trials). For the Segmentation trials, we created 12 correct test scenes that had a single bigram from familiarization displayed on the grid and 12 incorrect test scenes that had the characters in novel combinations not seen during familiarization, also presented on the grid individually (see Figure 3). During a test trial, a pair of test scenes (one correct and one incorrect) were presented successively for 4 s each (counterbalanced for order). Each correct and incorrect scene was used twice during the testing phase, thereby resulting in a total of 24 test trials. After viewing each test pair, participants were asked to choose which one belonged to the video they viewed during familiarization.

**FIGURE 4:**
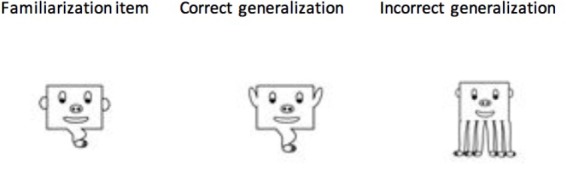
Example of Generalization and Identification test trials. For the Generalization test, participants viewed correct and incorrect generalizations. For the Identification trials, participants viewed familiarization items and incorrect generalization. Only a single image was displayed for each test trial.

For the Categorization trials, we had two subtests. The first subtest (Generalization trials) consisted of six correct generalizations and 6 incorrect generalizations. The correct generalizations were shape-based characters that maintained the correlated features of that set but also contained uncorrelated features that had not been seen during familiarization. By contrast, incorrect generalizations were novel characters whose configuration violated the correlated features of the set (see Figure 4). For example, a correct generalization might change the shape of the eyes within a character (i.e., a freely varying feature of the category), whereas an incorrect generalization would change the number of legs for a given character (a feature that was integral for category membership). The second subset (Identification trials) was comprised of 6 characters that had been seen during familiarization and 6 novel incorrect generalizations. Participants viewed each character in isolation during this test phase (for both Generalization and Identification trials). For Generalization trials, participants were asked to determine whether the character could have been part of the characters seen during familiarization, and for the Identification trials they were asked whether the character was part of the characters seen during familiarization. The Generalization trials always occurred first, as they tested whether the category contingency was learned, whereas the Identification trials verified they could remember the actual items from familiarization.

## Procedure

Participants were seated in a designated testing room and informed that they would be viewing a short video followed by some questions about what they saw. They did not receive any further details regarding the experimental design. All instructions for the functionally monolingual participants at Pennsylvania State University were in English while the bilingual participants at both locations received instructions in Spanish.

Participants viewed one of the two 4-min streams. Half of the participants viewed the stream created from Set A characters while the other half viewed the stream created from Set B characters. Following the 4-min stream, they received 24 Segmentation Trials, followed by 24 Categorization Trials. Upon completing the test trials, participants performed the Operational Span Task (OSPAN, Christoffels et al., 2006), a task testing working memory abilities. Participants were presented with a math problem (e.g., 2^∗^5+1 = 10) and were asked to indicate (within 3500 ms) whether it was correct. Subsequently, they were shown a word, and asked to remember it. After each set of trials (between 2 and 6), participants were asked to recall the words seen during that set. Accuracy on any given trial is determined by whether the participant correctly recalled a word after correctly classifying the math problem. All participants completed the OSPAN in the language of testing (English for monolinguals and Spanish for both bilingual groups) and the words used in each language were matched for frequency. After completing the OSPAN, participants filled out a Language History Questionnaire (Li et al., 2006).

## Results

We first established that the three groups did not differ in their working memory abilities. OSPAN scores did not differ across the three groups [monolingual mean = 42.83 (*SD* = 9.77), Spanish-Catalan bilingual mean = 38.22 (11.17), Spanish-English bilingual mean = 42.5 (10.05), one way ANOVA, *F*(2,68) = 1.45, *p* > 0.24]. All groups performed above chance on the Segmentation test, monolinguals: *t*(23) = 6.16, *p* < 0.001, Spanish-Catalan bilinguals: *t*(22) = 3.72, *p* = 0.001, Spanish-English bilinguals: *t*(23) = 3.97, *p* < 0.001 (see Table 1 for descriptive statistics). We divided the Categorization trials into two sets, as discussed in the methodology section above: Generalization trials and Identification trials. We calculated d’ (standardized hit rate - standardized false alarm rate) for participants for each of these two sets of trials. As is traditional in signal detection theory, a d’ of 0 would imply that participants performed at chance and were not sensitive to the differences between correct generalizations and false alarms. All three groups exhibited above chance sensitivity on both parts of the test. Monolinguals’ Generalization d’ score was 1.61 (*SD* = 0.81), *t*(23) = 9.7, *p* < 0.001; and their Identification d’ score was 1.24 (0.9), *t*(23) = 6.78, *p* < 0.001. Spanish-Catalan bilinguals’ Generalization d’ score was 1.5 (1.17), *t*(22) = 6.15, *p* < 0.001; and their Identification d’ score was 1.5 (0.89), *t*(22) = 8.09, *p* < 0.001. Spanish-English bilinguals’ Generalization d’ score was 1.19 (0.95), *t*(23) = 6.15, *p* < 0.001; and their Identification d’ score was 1.33 (1.1), *t*(23) = 5.91, *p* < 0.001.

**Table 1:** Descriptive statistics for each test type for monolinguals and bilinguals.

	Segmentation (out of 24)	Generalization (out of 12)	Identification (out of 12)
Monolinguals	16.2 (3.35)	9.45 (1.72)	8.67 (1.9)
Spanish-Catalan Bilinguals	15.74 (4.8)	9.22 (2.5)	9.22 (1.91)
Spanish-English Bilinguals	16.25 (5.2)	8.58 (2.0)	8.75 (2.3)


We further analyzed the data using a linear regression with Segmentation performance as the dependent variable, language background (monolingual, Spanish-Catalan bilingual, Spanish-English bilingual) as a between-subjects factor, and OSPAN scores as a covariate. The effects of group [*F*(2,67) = 0.09, *p* = 0.91] and the effect of OSPAN scores [*F*(1,67) = 0.29, *p =* 0.59] were not significant. For the categorization trials, we constructed a linear model with d’ scores as the dependent variable, language background (monolingual, Spanish-Catalan bilingual, Spanish-English bilingual) as a between-subjects factor, test type (Generalization and Identification) as a within-subjects factor, and OSPAN scores as a covariate. The effect of language background was not significant [*F*(2,135) = 0.76, *p* = 0.47], nor was the effect of test type [*F*(1,135) = 0.23, *p* = 0.63] nor the interaction between language background and test type [*F*(2,135) = 0.86, *p* = 0.42]. There was, however, a significant effect of OSPAN [*F*(1,135) = 4.3, p = 0.04, *η^2^* = 0.03]. Follow-up comparisons revealed that this effect was driven by the Spanish-Catalan bilinguals, whose OSPAN scores were strongly correlated with d’ scores (*r* = 0.35, *p* = 0.019), whereas the other two groups did not exhibit significant correlations (monolinguals: *r* = 0.1, *p* = 0.5; Spanish-English bilinguals: *r* = 0.05, *p* = 0.71).

As this task provided learners with the opportunity to track two separate types of co-occurrences, we checked to see whether the Segmentation and Generalization scores were correlated. A correlation between the Segmentation and Generalization test revealed that these two tests were significantly correlated, *r* = 0.32, *p* = 0.006. This effect was driven by the Spanish-English bilingual group (*r* = 0.73, *p* < 0.001), and was not present for the Spanish-Catalan bilinguals (*r* = 0.06, *p* = 0.8) nor the monolinguals (*r* = 0.15, *p* = 0.49).

## General Discussion

We tested how monolingual and bilingual learners contend with a multi-level statistical learning task. Three groups of participants were exposed to one of two streams that afforded learning of two types of co-occurrence regularities: one regularity based on the spatial relationship between characters and the other on learning feature-based associative relationships across each of the characters. Our results indicate that learners can extract multiple types of independent co-occurrence regularities within a single familiarization session and that performance on our task does not differ based on language background (i.e., monolingual versus bilingual).

To the best of our knowledge, this is the first study to directly investigate whether learners can track an input that affords learning two unrelated statistical dependencies. Previous research has found that both infant and adult learners can track multiple types of statistics from a single input when they are hierarchically organized, such that learning of one regularity is contingent on the other. For example, Saffran and Wilson (2003) familiarized 12-month-old infants to an artificial language constructed from a finite state grammar and found they could segment the speech stream and subsequently learn the underlying grammatical categories. Similarly, Kovács and Endress (2014) showed that 7-month-old infants could learn hierarchically ordered sets of stimuli. In the visual modality, adult learners are able to extract temporal statistics at both the local and global levels, also within a hierarchy (Jun and Chong, 2016). Our results suggest that statistical learning of multiple regularities does not necessarily rely on this hierarchical contingency and that multiple types of regularities may be tracked at once. While there have been multimodal tasks that afford this type of learning (e.g., Mitchel and Weiss, 2011), they rely on performing the exact same underlying computation on each input (i.e., tracking temporal transitional probabilities of triplets in the visual and auditory modality). Thus, our results extend our knowledge regarding statistical learning capacities. Specifically, we demonstrate that unrelated visual co-occurrence regularities can be successfully tracked over a single familiarization period.

Our findings also contribute to our understanding of how bilingualism impacts statistical learning. As noted in the Introduction, several studies have reported that statistical learning abilities appear to be equivalent in monolinguals and bilinguals (e.g., Yim and Rudoy, 2013; Bulgarelli and Weiss, 2016), though differences may emerge with multiple mappings (e.g., Poepsel and Weiss, 2016) or multiple cues available for segmentation (e.g., Bartolotti et al., 2011; Wang and Saffran, 2014). Further, visual statistical learning has been shown to be predictive of language learning outcomes in late L2 adult learners (Frost et al., 2013). The equivalent performance across two groups of bilingual learners with different linguistic backgrounds and monolingual learners lends further support to the notion that core abilities may be unchanged by language experience. Of course, in principle it is still possible that differences between groups might emerge if the parameters of the task were manipulated further (e.g., making the task more complex). Further, we did not test how the learning of co-occurrence regularities might have generalized to new characters and it is possible that differences could emerge with respect to the types of generalizations each group would permit. Thus, there are several future directions for this research program to explore.

**FIGURE 5:**
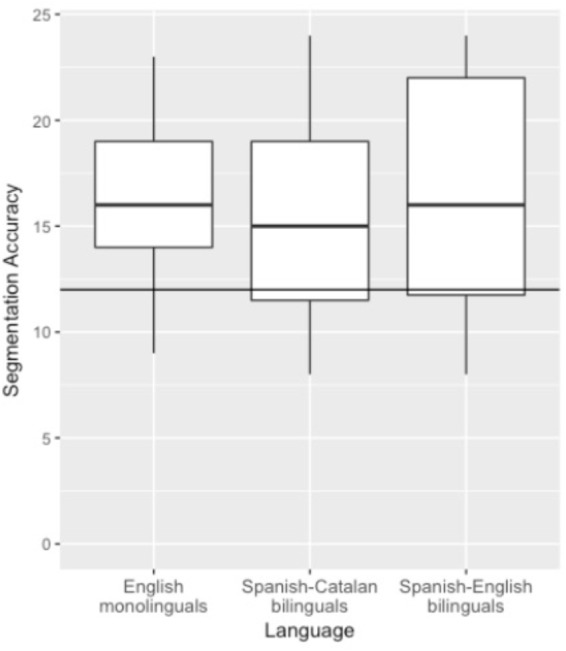
Distribution of performance on the Segmentation test across groups. The upper and lower hinges correspond to the first and third quartile. Horizontal line indicates chance.

Despite similar performance across groups, there were two subtle differences worth noting. One difference is that working memory performance was related to categorization performance for the Spanish-Catalan bilingual group, though not observed in the other groups. This discrepancy may have arisen due to the overall poorer performance on the working memory task by the Spanish-Catalan bilingual group, which was also characterized by greater variability. Another difference to note with respect to Spanish-English bilinguals is that their performance on the Segmentation and Generalization tasks were highly correlated, unlike the other groups. The Spanish-English bilinguals had the most variability in their performance on the Segmentation task (see Figure 5). The differences between the Spanish-English bilinguals and the other groups did not result in overall performance differences, but nonetheless raise the possibility that the Spanish-English bilinguals (or some subset thereof) engaged in different processing strategies relative to the other groups. We note that these subtle differences across bilingual groups could also be a result of differences in language background and experience. For example, the Spanish-Catalan bilingual group reported moderate proficiency in a language besides Spanish and Catalan. In some instances, research has found performance differences in language learning tasks between bilinguals and trilinguals (e.g., Dijkstra and van Hell, 2003; Byers-Heinlein and Werker, 2009). In addition to differences in L3 learning and proficiency, bilingualism is not a categorical variable (Luk and Bialystok, 2013), and bilinguals also vary in age of acquisition and language use, among other factors. While outside of the scope of the current study, we suggest that it would be fruitful to revisit these differences by further testing larger samples of bilinguals and also trilinguals, including those that differ in age of acquisition and the relationship between the known languages.

We close by noting the challenges ahead for research in this area. On the one hand, our study is consistent with other studies suggesting that bilingualism does not fundamentally alter statistical learning abilities (Bogulski, 2013; Yim and Rudoy, 2013; Bulgarelli and Weiss, 2016) in the absence of needing to remap words or suppress cues (Bartolotti et al., 2011; Poepsel and Weiss, 2016). Notwithstanding, we noted there are studies demonstrating that bilingual infants may be advantaged in tracking one or more patterns relative to monolinguals (Antovich and Graf Estes, 2017) and that visual statistical learning predicts successful morphological learning of non-native words in late L2 learning (Frost et al., 2013). In our view, the likely outcome from future work will bolster the analogy between statistical learning and a much older literature on learning to learn (e.g., Krechevsky, 1932; Dufort et al., 1954). This latter area of study finds that when learners are exposed to reward contingencies that frequently change, they become more sensitive to future changes (see Gallistel et al., 2001). Critically, though, the fundamental principles of learning remain unchanged (Gallistel et al., 2001; see also Poepsel and Weiss, 2016). So too, we believe the results here lend further support to the idea that bilingual experience alone does not alter performance in most statistical learning tasks. Rather, in limited circumstances (such as word learning), it may alter assumptions about the types of mappings afforded by the input (Byers-Heinlein and Werker, 2009; Poepsel and Weiss, 2016). Future work will need to more directly test how the cognitive consequences of bilingualism, such as enhanced inhibitory control, interface with statistical learning in order to generate a more nuanced understanding of whether the consequences of knowing more than one language can produce lasting effects for learning.

## Data Availability Statement

The dataset and analysis script for this study can be found at: https://osf.io/q57j9/?view_only=969ee2fa09eb4edbb542e0e33d41375a.

## Ethics Statement

This study was carried out in accordance with the recommendations of the Pennsylvania State University Institutional Review Board and the Universitat de Barcelona Institutional Review Board with written informed consent from all subjects. All subjects gave written informed consent in accordance with the Declaration of Helsinki. The protocol was approved by the IRB at both institutions.

## Author Contributions

FB, DW, and LB designed the experiment. FB created the stimuli, programmed the experiments, and conducted the analyses. FB and LB collected the data. All authors contributed to the write up.

## Conflict of Interest Statement

The authors declare that the research was conducted in the absence of any commercial or financial relationships that could be construed as a potential conflict of interest.

## Footnotes

1 We had intended to have a square and a rectangle shape, however the program rendered the square as a rectangle. Since it did not impact any of our questions of interest (i.e., these images were not involved in any critical manipulation), and due to the time sensitivity for the bilingual data collection, we chose to keep the rectangle. All analyses were conducted accordingly.

## References

[B1] Antovich D. M. Graf Estes K. (2017). Learning across languages: bilingual experience supports dual language statistical word segmentation. *Dev. Sci.* 21:e12548. 10.1111/desc.12548 28156032 PMC6594691

[B2] Bartolotti J. Marian V. Schroeder S. R. Shook A. (2011). Bilingualism and inhibitory control influence statistical learning of novel word forms. *Front. Psychol.* 2:324. 10.3389/fpsyg.2011.00324 22131981 PMC3223905

[B3] Bialystok E. (1999). Cognitive complexity and attentional control in the bilingual mind. *Child Dev.* 70 636–644. 10.1111/1467-8624.00046

[B4] Bialystok E. (2012). “The impact of bilingualism on language and literacy development,” in *The Handbook of Bilingualism and Multilingualism: Second Edition*, eds Bhatia T. K. Ritchie W. C. (Hoboken, NJ: Wiley-Blackwell Publishing), 624–648. 10.1002/9781118332382.ch25

[B5] Bialystok E. Craik F. I. M. Ruocco A. C. (2006). Dual-modality monitoring in a classification task: the effects of bilingualism and ageing. *Q. J. Exp. Psychol.* 59 1968–1983. 10.1080/17470210500482955 16987784

[B6] Bogulski C. A. (2013). *Are Bilinguals Better Learners? A Neurocognitive Investigation of the Bilingual Advantage*. State College, PA: The Pennsylvania State University.

[B7] Bulgarelli F. Lebkuecher A. L. Weiss D. J. (2018). Statistical learning and bilingualism. *Lang. Speech Hear. Serv. Sch.* 49 740–753. 10.1044/2018_LSHSS-STLT1-17-0139 30120450

[B8] Bulgarelli F. Weiss D. J. (2016). Anchors aweigh: the impact of overlearning on entrenchment effects in statistical learning. *J. Exp. Psychol. Learn. Mem. Cogn.* 42 1621–1631. 10.1037/xlm0000263 26950492 PMC5014725

[B9] Byers-Heinlein K. Werker J. F. (2009). Monolingual, bilingual, trilingual: infants’ language experience influences the development of a word-learning heuristic. *Dev. Sci.* 12 815–823. 10.1111/j.1467-7687.2009.00902.x 19702772

[B10] Christoffels I. K. de Groot A. M. B. Kroll J. F. (2006). Memory and language skills in simultaneous interpreters: the role of expertise and language proficiency. *J. Mem. Lang.* 54 324–345. 10.1016/J.JML.2005.12.004

[B11] Dijkstra T. van Hell J. G. (2003). Testing the language mode hypothesis using trilinguals. *Int. J. Biling. Educ. Biling.* 6 2–16. 10.1080/13670050308667769

[B12] Dufort R. H. Guttman N. Kimble G. A. (1954). One-trial discrimination reversal in the white rat. *J. Comp. Physiol. Psychol.* 47 248–249. 10.1037/h0057856 13163264

[B13] Escudero P. Mulak K. E. Fu C. S. L. Singh L. (2016). More limitations to monolingualism: bilinguals outperform monolinguals in implicit word learning. *Front. Psychol.* 7:1218. 10.3389/fpsyg.2016.01218 27574513 PMC4983614

[B14] Festman J. Rodriguez-Fornells A. Münte T. F. (2010). Individual differences in control of language interference in late bilinguals are mainly related to general executive abilities. *Behav. Brain Funct.* 6:5. 10.1186/1744-9081-6-5 20180956 PMC2830994

[B15] Fiser J. Aslin R. N. (2001). Unsupervised statistical learning of higher-order spatial structures from visual scenes. *Psychol. Sci.* 12 499–504. 10.1111/1467-9280.00392 11760138

[B16] Fiser J. Aslin R. N. (2005). Encoding multielement scenes: statistical learning of visual feature hierarchies. *J. Exp. Psychol. Gen.* 134 521–537. 10.1037/0096-3445.134.4.521 16316289

[B17] Frost R. Siegelman N. Narkiss A. Afek L. (2013). What predicts successful literacy acquisition in a second language? *Psychol. Sci.* 24 1243–1252. 23698615 10.1177/0956797612472207 PMC3713085

[B18] Gallistel C. R. Mark T. A. King A. P. Latham P. E. (2001). The rat approximates an ideal detector of changes in rates of reward: implications for the law of effect. *J. Exp. Psychol. Anim. Behav. Process.* 27 354–372. 10.1037/0097-7403.27.4.354 11676086

[B19] Gebhart A. L. Newport E. L. Aslin R. N. (2009). Statistical learning of adjacent and nonadjacent dependencies among nonlinguistic sounds. *Psychon. Bull. Rev.* 16 486–490. 10.3758/PBR.16.3.486 19451373 PMC2799498

[B20] Goetz P. J. (2003). The effects of bilingualism on theory of mind development. *Biling. Lang. Cogn.* 6 1–15. 10.1017/S1366728903001007 18329322

[B21] Gomez R. L. Gerken L. (1999). Artificial grammar learning by 1-year-olds leads to specific and abstract knowledge. *Cognition* 70 109–135. 10.1016/S0010-0277(99)00003-7 10349760

[B22] Greenberg A. Bellana B. Bialystok E. (2013). Perspective-taking ability in bilingual children: extending advantages in executive control to spatial reasoning. *Cogn. Dev.* 28 41–50. 10.1016/j.cogdev.2012.10.002 23486486 PMC3593058

[B23] Jun J. Chong S. C. (2016). Visual statistical learning of temporal structures at different hierarchical levels. *Attent. Percept. Psychophys.* 78 1308–1323. 10.3758/s13414-016-1104-9 27068052

[B24] Kovács A. M. Mehler J. (2009a). Cognitive gains in 7-month-old bilingual infants. *Proc. Natl. Acad. Sci. U.S.A.* 106 6556–6560. 10.1073/pnas.0811323106 19365071 PMC2672482

[B25] Kovács A. M. Mehler J. (2009b). Flexible learning of multiple speech structures in bilingual infants. *Science* 325 611–612. 10.1126/science.1173947 19589962

[B26] Kovács ÁM. Endress A. D. (2014). Hierarchical processing in seven-month-old infants. *Infancy* 19 409–425. 10.1111/infa.12052

[B27] Krechevsky I. (1932). “Hypotheses” in rats. *Psychol. Rev.* 39 516–532. 10.1037/h0073500

[B28] Li P. Sepanski S. Zhao X. (2006). Language history questionnaire: a Web-based interface for bilingual research. *Behav. Res. Methods* 38 202–210. 10.3758/BF03192770 16956095

[B29] Luk G. Bialystok E. (2013). Bilingualism is not a categorical variable: interaction between language proficiency and usage. *J. Cogn. Psychol.* 29 997–1003. 10.1080/20445911.2013.795574 PMC3780436 24073327

[B30] Maye J. Werker J. F. Gerken L. (2002). Infant sensitivity to distributional information can affect phonetic discrimination. *Cognition* 82 B101–B111. 10.1016/S0010-0277(01)00157-3 11747867

[B31] Mitchel A. D. Weiss D. J. (2011). Learning across senses: cross-modal effects in multisensory statistical learning. *J. Exp. Psychol. Learn. Mem. Cogn.* 37 1081–1091. 10.1037/a0023700 21574745 PMC4041380

[B32] Moradzadeh L. Blumenthal G. Wiseheart M. (2015). Musical training, bilingualism, and executive function: a closer look at task switching and dual-task performance. *Cogn. Sci.* 39 992–1020. 10.1111/cogs.12183 25289704

[B33] Paap K. R. Greenberg Z. I. (2013). There is no coherent evidence for a bilingual advantage in executive processing. *Cogn. Psychol.* 66 232–258. 10.1016/j.cogpsych.2012.12.002 23370226

[B34] Park J. S. Miller C. A. Rosenbaum D. A. Sanjeevan T. van hell J. G. Weiss D. J. (2017). Bilingualism and procedural learning in typically developing children. *J. Speech Lang. Hear. Res.* 61 634–644. 10.1044/2017_JSLHR-L-16-0409 29466557

[B35] Poarch G. J. van Hell J. G. (2012). Executive functions and inhibitory control in multilingual children: evidence from second-language learners, bilinguals, and trilinguals. *J. Exp. Child Psychol.* 113 535–551. 10.1016/J.JECP.2012.06.013 22892367

[B36] Poepsel T. J. Weiss D. J. (2016). The influence of bilingualism on statistical learning with multple inputs. *Cognition* 152 9–19. 10.1016/j.cognition.2016.03.001 27015348

[B37] Reber A. S. (1967). Implicit learning of artificial grammars. *J. Verbal Learn. Verbal Behav.* 6 855–863. 10.1016/S0022-5371(67)80149-X

[B38] Reeder P. A. Newport E. L. Aslin R. N. (2013). From shared contexts to syntactic categories: the role of distributional information in learning linguistic form-classes. *Cogn. Psychol.* 66 30–54. 10.1016/j.cogpsych.2012.09.001 23089290 PMC3621024

[B39] Saffran J. R. Aslin R. N. Newport E. L. (1996a). Statistical learning by 8-month-old infants. *Science* 274 1926–1928. 8943209 10.1126/science.274.5294.1926

[B40] Saffran J. R. Newport E. L. Aslin R. N. (1996b). Word segmentation: the role of distributional cues. *J. Mem. Lang.* 35 606–621. 10.1006/jmla.1996.0032

[B41] Saffran J. R. Wilson D. P. (2003). From syllables to syntax: multilevel statistical learning by 12-month-old infants. *Infancy* 4 273–284. 10.1207/S15327078IN0402_07

[B42] Sebastián-Gallés N. Albareda-castellot B. Weikum W. M. Werker J. F. (2012). A bilingual advantage in visual language discrimination in infancy. *Psychol. Sci.* 23 994–999. 10.1177/0956797612436817 22810164

[B43] Wang T. Saffran J. R. (2014). Statistical learning of a tonal language: the influence of bilingualism and previous linguistic experience. *Front. Psychol.* 5:953. 10.3389/fpsyg.2014.00953 25232344 PMC4153027

[B44] Weiss D. J. Gerfen C. Mitchel A. D. (2009). Speech segmentation in a simulated bilingual environment: a challenge for statistical learning? *Lang. Learn. Dev.* 5 30–49. 10.1080/15475440802340101 24729760 PMC3981102

[B45] Weiss D. J. Poepsel T. J. Gerfen C. (2015). “Tracking multiple inputs: the challenge of bilingual statistical learning,” in *Implicit and Explicit Learning of Languages*, ed. Rebuschat P. (Amsterdamn: John Benjamins Publishing Company), 167–190. 10.1075/sibil.48.08wei

[B46] Yim D. Rudoy J. (2013). Implicit statistical learning and language skills in bilingual children. *J. Speech Lang. Hear. Res.* 56 310–322. 10.1044/1092-4388(2012/11-0243)b 22896046

[B47] Yu C. Smith L. B. (2007). Rapid word learning under uncertainty via cross-situational statistics. *Psychol. Sci.* 18 414–420. 10.1111/j.1467-9280.2007.01915.x 17576281

